# The Automated External Defibrillator: Heterogeneity of Legislation, Mapping and Use across Europe. New Insights from the ENSURE Study

**DOI:** 10.3390/jcm10215018

**Published:** 2021-10-28

**Authors:** Enrico Baldi, Niccolò B. Grieco, Giuseppe Ristagno, Hajriz Alihodžić, Valentine Canon, Alexei Birkun, Ruggero Cresta, Diana Cimpoesu, Carlo Clarens, Julian Ganter, Andrej Markota, Pierre Mols, Olympia Nikolaidou, Martin Quinn, Violetta Raffay, Fernando Rosell Ortiz, Ari Salo, Remy Stieglis, Anneli Strömsöe, Ingvild Tjelmeland, Stefan Trenkler, Jan Wnent, Jan-Thorsten Grasner, Bernd W. Böttiger, Simone Savastano

**Affiliations:** 1 Department of Molecular Medicine, Section of Cardiology, University of Pavia, 27100 Pavia, Italy; 2 Cardiac Intensive Care Unit, Arrhythmia and Electrophysiology and Experimental Cardiology, Fondazione IRCCS Policlinico San Matteo, 27100 Pavia, Italy; 3 Italian Resuscitation Council, 40128 Bologna, Italy; niccolo.grieco@gmail.com (N.B.G.); gristag@gmail.com (G.R.); s.savastano@smatteo.pv.it (S.S.); 4 Cardiology Department, Niguarda Hospital, 20162 Milan, Italy; 5 Department of Pathophysiology and Transplantation, University of Milan, 20122 Milan, Italy; 6 Emergency Medical Service, Public Institution Health Centre ‘Dr. Mustafa Šehović’ and Faculty of Medicine, University of Tuzla, 75000 Tuzla, Bosnia and Herzegovina; hajriz.a@hotmail.com; 7 CHU Lille, ULR 2694-METRICS: Évaluation des Technologies de Santé et des Pratiques Médicales, University of Lille, F-59000 Lille, France; valentine.baert@univ-lille.fr; 8 French National Out-of-Hospital Cardiac Arrest Registry-Registre Électronique des Arrêts Cardiaques, F-59000 Lille, France; 9 Medical Academy Named after S. I. Georgievsky of V. I. Vernadsky Crimean Federal University, 95000 Simferopol, Russia; birkunalexei@gmail.com; 10 Quality and Research Division, Federazione Cantonale Ticinese Servizi Ambulanza (FCTSA), 6500 Bellinzona, Switzerland; ruggero.cresta@fctsa.ch; 11 Fondazione Ticino Cuore, 6900 Lugano, Switzerland; 12 Emergency Department, Faculty of Medicine, “Grigore T. Popa” University of Medicine and Pharmacy, 700115 Iasi, Romania; dcimpoiesu@yahoo.com; 13 Luxembourg Resuscitation Council, 2680 Luxembourg, Luxembourg; carlo.clarens@lrc.lu; 14 Department of Cardiovascular Surgery, Faculty of Medicine, University Heart Center Freiburg, 79085 Freiburg, Germany; julian.ganter@uniklinik-freiburg.de; 15 Slovenian Resuscitation Council, Slovenian Society of Emergency Medicine, 1000 Ljubljana, Slovenia; andrej.markota@ukc-mb.si; 16 Medical Intensive Care Unit, University Medical Centre Maribor, 2000 Maribor, Slovenia; 17 Service des Urgences et du SMUR, CHU Saint-Pierre, Université Libre de Bruxelles, 1000 Bruxelles, Belgium; pierre.mols@gmail.com; 18 EMS—National Center for Emergency Care, 546 Thessaloniki, Greece; olynmed@yahoo.gr; 19 Out-of-Hospital Cardiac Arrest Registry Steering Group, National University of Ireland, H91 CF50 Galway, Ireland; martin.quinn@hse.ie; 20 Department of Medicine, European University Cyprus, Nicosia 2404, Cyprus; violetta.raffay@gmail.com; 21 Serbian Resuscitation Council, 21102 Novi Sad, Serbia; 22 Servicio de Urgencias Médicas 061 La Rioja, 26007 Logroño, Spain; fernando.rosell@juntadeandalucia.es; 23 Department of Emergency Medicine and Services, University of Helsinki and Helsinki University Hospital, 00530 Helsinki, Finland; ari.salo@hus.fi; 24 Department of Cardiology, Amsterdam University Medical Center, Location AMC, 1105 Amsterdam, The Netherlands; r.stieglis@amc.uva.nl; 25 School of Education, Health and Social Studies, Dalarna University, S-79188 Falun, Sweden; ase@du.se; 26 Centre for Clinical Research Dalarna, Uppsala University, S-79182 Falun, Sweden; 27 Department of Prehospital Care, Region of Dalarna, S-79129 Falun, Sweden; 28 Division of Prehospital Services, Oslo University Hospital, 0372 Oslo, Norway; ingvild@nakos.no; 29 Faculty of Medicine, Institute of Clinical Medicine, University of Oslo, 0372 Oslo, Norway; 30 Institute for Emergency Medicine, University Hospital Schleswig-Holstein, 24105 Kiel, Germany; wnent@eureca-two.eu (J.W.); jan-thorsten.graesner@uksh.de (J.-T.G.); 31 Department of Anaesthesiology and Intensive Medicine, Medical Faculty, P.J. Safarik University, 040 11 Kosice, Slovakia; strenkler@gmail.com; 32 Department of Anesthesiology, University Hopspital Schleswig-Holstein, Campus Kiel, 24105 Kiel, Germany; 33 School of Medicine, University of Namibia, Windhoek 10005, Namibia; 34 Department of Anaesthesiology and Intensive Care Medicine, University Hospital and Medical Faculty of Cologne, 50931 Cologne, Germany; bernd.boettiger@uk-koeln.de; 35 European Resuscitation Council (ERC), 2845 Niel, Belgium; 36 Division of Cardiology, Fondazione IRCCS Policlinico San Matteo, 27100 Pavia, Italy

**Keywords:** out-of-hospital cardiac arrest (OHCA), automated external defibrillator (AED) system, legislation, first responders

## Abstract

Introduction: The rapid use of an automated external defibrillator (AED) is crucial for increased survival after an out-of-hospital cardiac arrest (OHCA). Many factors could play a role in limiting the chance of an AED use. We aimed to verify the situation regarding AED legislation, the AED mapping system and first responders (FRs) equipped with an AED across European countries. Methods: We performed a survey across Europe entitled “European Study about AED Use by Lay Rescuers” (ENSURE), asking the national coordinators of the European Registry of Cardiac Arrest (EuReCa) program to complete it. Results: Nineteen European countries replied to the survey request for a population covering 128,297,955 inhabitants. The results revealed that every citizen can use an AED in 15 countries whereas a training certificate was required in three countries. In one country, only EMS personnel were allowed to use an AED. An AED mapping system and FRs equipped with an AED were available in only 11 countries. The AED use rate was 12–59% where AED mapping and FR systems were implemented, which was considerably higher than in other countries (0–7.9%), reflecting the difference in OHCA survival. Conclusions: Our survey highlighted a heterogeneity in AED legislation, AED mapping systems and AED use in Europe, which was reflected in different AED use and survival.

## 1. Introduction

Early cardiopulmonary resuscitation (CPR) and a rapid use of an automated external defibrillator (AED) are essential steps to improve survival after an out-of-hospital cardiac arrest (OHCA), as highlighted in the “Chain of Survival” [1,2]. Early defibrillation increases not only the rate of return of spontaneous circulation (ROSC) and survival but also a favorable neurological outcome at the hospital discharge, irrespective of it being a witnessed event, bystander CPR or an initial rhythm [3,4,5]. AED use by a bystander or first responders (FRs; i.e., police officers, firefighters, off-duty medical personnel and trained lay persons alerted when a patient experiences an OHCA nearby) before the arrival of emergency medical service (EMS) personnel is of a primary importance particularly when considering that the earlier the defibrillation, the higher the chance of survival [6,7,8]. This concept was proven in the early 2000s, when the public-access defibrillation (PAD) trial was carried out in the USA involving security officers after cardiac arrests in casinos [9] and has been continuously reinforced by numerous scientific publications subsequently released [10,11]. A recent meta-analysis of six observational studies, without a critical risk of bias, confirmed that bystander AED use was associated with a higher survival to the hospital discharge (all rhythms OR: 1.73 (95% CI: 1.36, 2.18); shockable rhythms OR: 1.66 (95% CI: 1.54, 1.79)) and a favorable neurological outcome (all rhythms OR: 2.12 (95% CI: 1.36, 3.29); shockable rhythms OR: 2.37 (95% CI: 1.58, 3.57)) [12].

Considering that no particular skills are needed to correctly use an AED [13] and no risks for rescuers are present [14,15], the guidelines have highlighted from 2015 onwards [16,17] that the use of an AED even by untrained lay persons must be encouraged.

However, despite the well-established favorable role on survival and the resulting guideline recommendations, the rate of AED use is quite heterogenous among the different countries worldwide, including in Europe [18] where it is very low in many countries. Many factors could play a role in limiting the chance of AED use such as the availability of the AED, the presence of an FR alerting system and also local laws regulating the use of an AED by a bystander [19].

Therefore, we aimed to verify the situation regarding the legislation related to the use of an AED, an AED mapping system and the eventual dispatch of a first responder equipped with an AED across European countries. We also aimed to assess the eventual differences in the rates of AED use and the outcomes of patients among the countries according to the different organizational and legislation settings.

## 2. Materials and Methods

We performed a survey across Europe entitled “European Study About AED Use by Lay Rescuers” (ENSURE) asking primarily the 29 national coordinators of the European Registry of Cardiac Arrest (EuReCa) [20] program to fill in the questionnaire, referring to the EuReCa TWO period (October 2017–December 2017). The study was endorsed by the European Resuscitation Council (ERC) Research NET and by the Italian Resuscitation Council (IRC).

The questionnaire was divided into different parts (Supplementary File S1). The first part comprised general questions regarding the type of registry and the population covered; the second part focused on real-life data about OHCAs collected during the EuReCa TWO period and the third part queried the organizational and legislation setting both during the study period and at the time of survey completion (mid-2020). Considering the nature of the study, no further ethics approval was needed.

## 3. Results

**Figure 1:**
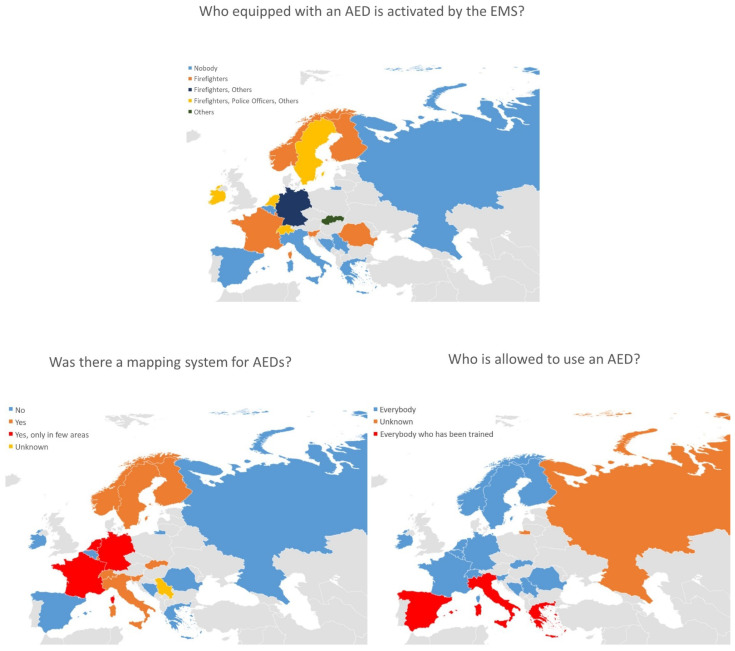
Graphical representation across the different European countries regarding who is, equipped with an AED, activated by the EMS, if there was an AED mapping system and who is allowed to use an AED.

We received replies from 19 European countries out of the 29 invited. The answers referred to 10 national registries and 9 regional or provincial registries for a total population of about 128,297,955 inhabitants. Regarding AED legislation, in 15 out of 19 countries, every citizen was allowed to use an AED whereas a training certificate was required in three countries. In one country, only EMS personnel were allowed to use an AED (Table 1 and Figure 1). An AED mapping system covering the whole nation was available in only 8 out of 19 countries and it was available only in certain areas in 3 out of 19 countries. Concerning the dispatch of first responders equipped with an AED, this was available in 11 out of 19 countries (Figure 1).

The data regarding the number of OHCA occurred, the use of an AED before EMS arrival, the ROSC and survival across the different european countries during the EuReCa TWO period (October 2017–December 2017) are presented in Table 2.

## 4. Discussion

Our study has presented data regarding the legislations regulating AED use, the AED mapping systems and AED use across Europe, highlighting a great heterogeneity across European countries. This is reflected in important variations in AED use and OHCA patient survival in different countries.

### 4.1. The Importance of the Legislation Rule

It is reasonable to assume that the rate of AED use is influenced by the type of legislation regulating the use of an AED by lay persons [19] although evidence in the literature on this topic is limited. Notably, in those countries where a certificate is needed for lay persons to be allowed to use an AED, the use of an AED is low (between 2.4% and 5%) [21,22] compared with those countries where a “Good Samaritan” law is in force and all citizens can freely use an AED if necessary (about 15–20% of an AED use before EMS arrival) [10,23,24,25]. Concerning Europe, the last survey about legislation rules on AED use dates back 11 years [26]. It highlighted that the use of an AED was allowed for all the citizens in one third of interviewed countries, for citizens with a certificate in another third and only for physicians or EMS personnel in the last third. Our study outlined that the situation has considerably improved in ten years: in about 80% of the countries, every citizen was allowed to use an AED. However, a training certificate was still required in three countries (Greece, Italy and Spain) and in one (Russia Federation) only EMS personnel were allowed to use an AED. Considering that six years have passed since the ERC 2015 guidelines were issued, we believe that the situation is quite alarming and suggests that several countries need to rapidly adhere to the recommendations allowing untrained bystanders to use an AED, thus increasing the rate of lay defibrillation and, therefore, the chance of survival of OHCA patients.

### 4.2. AED Mapping and First Responder Systems

The chance of receiving an early defibrillation depends on the availability of PADs [27]. Considering that the survival to discharge decreases for every minute of delay to defibrillation [17], it is crucial to retrieve an AED as soon as possible [28]. For this reason, the guidelines since 2015 have recommended that publicly accessible AEDs are registered and mapped so that dispatchers can direct CPR providers to the closest AED, optimizing the system response [29]. Our survey highlighted that an AED mapping system was available in just over half of the countries and this percentage dropped to only 40% if we considered only the countries with an AED mapping system available for the whole nation. No system mapping AEDs was available in 8 out of 19 countries. This aspect should be improved to increase the chance that an AED is brought to the scene in case of an OHCA. From a scientific point of view, the possibility of merging the location of the OHCA and the position of the AED by using both cardiac arrest registries and AED mapping systems could allow the development of new algorithms to relocate the AEDs with the intent to improve optimal coverage [30] to use more properly the AEDs already available without necessarily increasing their numbers as well as to exploit new innovative methods to get an AED to the scene such as the use of drones [31]. Furthermore, a crucial role in enhancing the possibility of providing early defibrillation is played by FRs [6,25,32,33,34]. FRs are commonly divided into “professional FRs” such as police officers, firefighters or off-duty medical personnel and “citizen FRs” meaning trained lay persons [7]. For this latter category, the only chance of using an AED depends on the availability of an AED on site and the possibility of receiving instructions from the dispatch center (or by the alerting APP) about the closest AED, stressing once again the importance of AED mapping. For the professional FRs, the situation is different because they are often equipped with an AED and can bring the defibrillator to the OHCA scene when alerted, resulting in a reduction of the time to the first defibrillation, which is associated with an increased chance of survival [35]. The effectiveness and the capability of the FR system to increase survival is already well-documented [8]. In our survey, we found that only in 11 out of 19 countries were the professional FRs alerted by the EMS equipped with an AED, stressing the need to improve this aspect throughout Europe.

### 4.3. Differences in AED Use, ROSC and Survival across Europe

Important differences among the countries regarding the rate of AED use before the arrival of the EMS have been already evidenced [18]. Our survey confirmed that in those countries where the law permits only trained bystanders to use an AED such as Greece, Italy and Spain, the chance of being defibrillated before the arrival of the EMS is particularly low (from 2.6 to 7%). However, the percentage of AED use before the arrival of the EMS appeared to be deeply varied across the European countries and it was low also in several countries where everybody is allowed to use an AED (e.g., Belgium, Ireland, Romania, Slovakia and Slovenia), suggesting that probably it is not only a matter of law. Our survey hinted that in countries where AED mapping and an FRs system are implemented alongside a permissive law, the rate of AED use was higher. This was particularly clear in several countries such as The Netherlands, Norway, Sweden and Switzerland, where systems to save a life were implemented many years ago [6,10,34]. In these countries, the rate of AED use was between 12 and 59%, considerably higher than in other countries (from 0 to 7.9%). All the above is perfectly in line with the latest ERC guidelines, which, for the very first time, have dedicated an entire chapter to the importance of the “Systems saving lives” [28], embracing the philosophy of the European Resuscitation Academy (ERA) and the Global Resuscitation Alliance (GRA) [36,37].

### 4.4. Limitations

Our study has limitations. The first is that we received data from 19 European countries; this does not represent all the countries present in Europe. However, considering that the registries included covered more than 125 million people, we believe that our study could be considered to be representative of the European situation. The second limitation is that we decided to refer, regarding OHCA data, to the EuReCa TWO period. This was chosen to consider the same time period in all the countries facilitating data retrieving and because a few registries collected data only during the EuReCa surveys, allowing an increase in the data collected for the present study. We decided to overcome this limitation by allowing the presentation of past data and to ask in the survey for the organizational and legislation setting both during the EuReCa TWO period and at the time of the survey completion (mid-2020). This allowed us to present data representing as much as possible the actual European situation. Considering this type of situation is constantly evolving, we marked the eventual subsequent changes that intervened after the survey completion, as seen in Table 1.

## 5. Conclusions

Our survey highlighted a great heterogeneity in terms of the legislations regulating the use of an AED, AED mapping systems and AED use across Europe, therefore limiting the OHCA survival in many European regions and countries. Considering the undoubted importance of all these three actions to maximize the chance of survival after an OHCA, we strongly suggest the following to all the European countries and their governments:

1. To issue a law that allow all citizens, including untrained ones, to use an AED in the case of a suspected OHCA and protecting them against any legal consequences.
2. To make an AED map compulsory that includes all public AEDs and that is linked to the emergency medical system dispatch center.
3. To implement FR systems, including both citizens trained in CPR and professional FRs (i.e., police officers, firefighters, off-duty medical personnel) possibly equipped with an AED, to increase the rate of defibrillation before the arrival of the EMS.
4. To unify cardiac arrest registries among European countries to harmonize data collection and better comprehend the European strategies to implement an improved OHCA survival.

## Figures and Tables

**Table 1:** AED legislation in different European countries updated in 2020 and the eventual differences compared with the EuReCa TWO period (late 2017).

Country	Is There Any Special Legislation on the Use of AEDs?	Who Is Allowed to Use an AED?	Has the Legislation Changed after December 2017?	Are There Legislation Differences in the Different Regions of Your Country?	Was There An aed Mapping System in Your Region/Country in the Study Period?	Did the (EMS) Activate FRS Equipped with an Aed in Your Region/Country in the Study Period?
Belgium	Yes	Everybody	No	No	No	No
Bosnia and Herzegovina	No	Everybody	No	No	No	No
Finland	No	Everybody	No	No	Yes	Yes
France	Yes	Everybody	Yes **	No	Yes, only in some areas	Yes
Germany	No	Everybody	No	No	Yes, only in a few areas	Yes, only in a few areas
Greece	Yes	Everybody trained *	No	No	No	No
Ireland	No	Everybody	No	No	No	Yes
Italy	Yes	Everybody trained	No ***	No	Yes	No
Luxembourg	Yes	Everybody	No	No	Yes	Yes
The Netherlands	No	Everybody	No	No	Yes, only in a few areas	Yes
Norway	No	Everybody	No	No	Yes	Yes, only in a few areas
Romania	Yes	Everybody	No	No	No	Yes
Russian Federation	No	EMS providers only	No	No	No	No
Serbia	No	Everybody	No	No	Unknown	No
Slovakia	No	Everybody	No	No	Yes	Yes
Slovenia	Yes	Everybody	No	No	Yes	Yes, only in a few areas
Spain	Yes	Everybody trained	No	No	No	No
Sweden	No	Everybody	No	No	Yes	Yes
Switzerland	No	Everybody	No	No	Yes	Yes

* In Greece, the law allows only trained lay persons to use an AED but it also establishes that anyone who offers immediate help in goodwill to a cardiac arrest victim cannot be prosecuted. ** A “Good Samaritan” law allowing all citizens to use an AED was issued in July 2020 in France. *** In Italy, the Parliament approved a “Good Samaritan” law in late July 2021, which is currently being implemented.

**Table 2:** Number of OHCAs, AED use, ROSCs and survival across the different European countries during the EuReCa TWO period (October 2017–December 2017).

Country	Regional or National Registry	Population Covered (n)	Name of the Registry	OHCAs with EMS Attempted Resuscitation Occurring in the Period Oct 2017–Dec 2017 (EuReCa TWO Period) (n)	OHCAs in the Utstein Comparator Group (UCG) * (n, %)	OHCAs in Which an AED Was Attached before EMS Arrival (n, %)	OHCAs in Which the AED Was Attached by Firefighters, Police Officers or Other FRs (n, %)	OHCAs in Which the AED Was Attached by Bystanders (n, %)	OHCAs in Which an AED Was Attached before EMS Arrival and a Shock Was Delivered (n)	OHCAs in Which the Shock Was Delivered by Firefighters, Police Officers or Other FRs (%)	OHCAs in Which the Shock Was Delivered by Bystanders (%)	ROSC in the UCG (n, %)	Survival at Discharge in the UCG (n, %)
Belgium	National	2,834,000	Belgian Cardiac Registry (B-CAR)	377	57 (15.1)	26 (6.9)	22 (84.6)	4 (15.4)	16 (4.2)	12 (75)	4 (25)	31 (54.4)	16 (28.1)
Bosnia and Herzegovina	Regional	110,979	Utstein Resuscitation Registry	22	1 (4.5)	0 (0)	N/A	N/A	0 (0)	N/A	N/A	0 (0)	0 (0)
Finland	Regional	655,395	Helsinki out-of-hospital cardiac arrest registry	76	19 (25)	6 (7.9)	N/A	N/A	3 (3.9)	N/A	N/A	13 (68)	7 (37)
France	National	17,833,002	Electronic Registry of Cardiac Arrests (RéAC)	2604	177 (6.8)	2066 (79.3) **	1874 (90.7)	192 (9.3)	418 (16)	368 (88)	50 (12)	95 (53.7)	40 (22.6)
Germany	National	26,600,000	Deutsches Reanimations Register (German Resuscitation Registry)	6969	524 (7.5)	0 (0)	N/A	N/A	0 (0)	N/A	N/A	372 (71)	30 (17)
Greece	Regional	1,500,000	National Center For Emergency Care (Northern Greece, Thessaloniki)	71	22 (31)	5 (7)	N/A	N/A	1 (1.4)	N/A	N/A	8 (36)	1 (4.6)
Ireland	National	4,757,976	Out-of-Hospital Cardiac Arrest Register (OHCAR)	594	72 (12.1)	21 (3.5)	17 (81)	4 (19)	21 (3.5)	15 (71.4)	6 (28.6)	28 (39)	18 (25)
Italy	Regional	545,810	Lombardia Cardiac Arrest Registry (LombardiaCARe)	152	17 (11.2)	4 (2.6)	0 (0)	4 (100)	2 (1.3)	0 (0)	2 (100)	9 (52.9)	3 (17.6)
Luxembourg	National	602,005	Cardlux	140	24 (17.1)	12 (8.6)	N/A	N/A	5 (3.6)	N/A	N/A	10 (41.6)	4 (16.6)
The Netherlands	Regional	2,578,552	Amsterdam Resuscitation Studies (ARREST)	314	81 (25.8)	183 (58.2)	146 (79.8)	37 (20.2)	74 (23.6)	52 (70)	22 (30)	5 (69)	33 (40.7)
Norway	National	5,336,494	Norwegian Cardiac Arrest Registry	848	95 (11.2)	103 (12.1)	57 (55.3)	46 (44.7)	30 (3.5)	N/A	N/A	56 (59)	42 (44.2)
Romania	National	4,086,753	Registrul Roman al Stopului Cardiac (Romanian Registry of Cardiac Arrest)	512	30 (5.8)	27 (5.3)	27 (100)	0 (0)	12 (2.3)	12 (100)	0 (0)	N/A	N/A
Russian Federation	Regional	1,913,731	Crimean Out-of-Hospital Cardiac Arrest and Resuscitation Registry (COHCARR) ***	N/A	N/A	N/A	N/A	N/A	N/A	N/A	N/A	N/A	N/A
Serbia	National	1,227,069	EuReCa Srbija	409	42 (10.3)	0 (0)	0 (0)	0 (0)	0 (0)	0 (0)	0 (0)	8 (19.1)	16 (38)
Slovakia	Other	3,300,000	Ad hoc for EuReCa TWO	693	93 (13.4)	6 (0.9)	N/A	N/A	2 (0.3)	N/A	N/A	45 (48.4)	24 (25.8)
Slovenia	Other	1,191,479	Ad hoc for EuReCa TWO	178	35 (19.7)	13 (7.3)	10 (76.9)	3 (23.1)	11 (6.2)	8 (73)	2 (18)	22 (63)	11 (31.4)
Spain	National	42,750,768	Out-of-Hospital Spanish Registry of Cardiac Arrest (OHSCAR)	2148	342 (15.9)	75 (3.5)	44 (58.7)	31 (41.3)	38 (1.8)	13 (34.2)	25 (65.8)	209 (61.1)	100 (29.2)
Sweden	National	10,120,242	The Swedish Registry of Cardiopulmonary Resuscitation (SRCR)	1466	171 (11.7)	336 (25)	269 (80)	44 (13.1)	84 (5.7)	46 (55)	27 (32)	96 (56.1)	101 (59.1)
Switzerland	Regional	353,700	Ticino Registry Cardiac Arrest (TiReCA)	53	9 (17)	19 (35.8)	12 (63.1)	7 (36.8)	2 (3.8)	1 (50)	1 (50)	4 (44.4)	2 (22.2)

AED: automated external defibrillator; OHCA: out-of-hospital cardiac arrest. * Utstein comparator group: bystander-witnessed for the first shockable rhythm. ** In France, the emergency medical system is by definition medicalized. Firefighters are trained in BLS and routinely alerted in case of an OHCA but they are considered not to be part of the EMS response and, therefore, are classified as first responders. *** The Crimean Out-of-Hospital Cardiac Arrest and Resuscitation Registry (COHCARR) started to collect data from 1 January 2018 onwards.

## Supplementary Materials

The following are available online at https://www.mdpi.com/article/10.3390/jcm10215018/s1, Supplementary File S1: ENSURE study questionnaire.
