# Supplementary material for: The Automated External Defibrillator: Heterogeneity of Legislation, Mapping and Use across Europe. New Insights from the ENSURE Study
Source: J Clin Med. 2021 Oct 28;10(21):5018. doi: 10.3390/jcm10215018 (PMC8585055; doi:10.3390/jcm10215018)
Supplement: Supplementary file 1 [file jcm-10-05018-s001.zip › jcm-1433813-supplementary.pdf]

# ENSURE Study

Questionnaire regarding AED use in all the european nations - EuropeaN Study about AED Use by layREscuers

*Campo obbligatorio

1. **Indirizzo email** *

2. **Your name** *

3. **Your country** *

## ENSURE Study - general questions

Questionnaire regarding AED use in all the european nations - EuropeaN Study about AED Use by layREscuers

4. **1. Is your registry regional-based or nation-wide?** *
   *Contrassegna solo un ovale.*
   - Regional
   - National
   - Other

5. **2. Which is the population covered by your registry? (number of citizen covered)** *

6. **3. Which is the name of your Registry?** *

## ENSURE Study - registry question

Please, refer to the period from October 1st, 2017 to December 31st, 2017

7. **4. How many out-of-hospital cardiac arrests (OHCAs) in which resuscitation was attempted occurred from October 1st, 2017 to December 31st, 2017 in your Registry?** *

8. **5. How many OHCAs were witnessed by EMS in that period? (absolute number)** *

9. **6. How many OHCAs were witnessed by bystander in that period? (absolute number)** *

10. **7. How many OHCAs were bystander-witnessed with a first shockable rhythm (Utstein Comparator Group)? (absolute number)** *

11. **8. In how many cases an AED was attached before EMS arrival in that period? (absolute number)** *

12. **8.1 (Only if available): In how many of these cases the AED was attached by first responder (firefighters / police officers or other first responders)? - Please indicate the percentage respect to the total number provided in question 8**

13. **8.2 (Only if available): In how many of these cases the AED was attached by laypeople? - Please indicate the percentage respect to the total number provided in question 8**

14. **9. In how many cases an AED was attached before EMS arrival and a shock was delivered in that period? (absolute number)** *

15. **9.1(Only if available): In how many of these cases the shock was delivered by first responder (firefighters / police officers or other first responders)? - Please indicate the percentage respect to the total number provided in question 9**

16. **9.2 (Only if available): In how many of these cases the shock was delivered by laypeople? - Please indicate the percentage respect to the total number provided in question 9**

17. **10. Which is the percentage of ROSC in the Utstein Comparator Group (OHCAs witnessed by bystander and with first rhythm shockable) in that period? (percentage)** *

18. **11. Which is the percentage of survival at discharge in the Utstein Comparator Group (OHCAs witnessed by bystander and with first rhythm shockable) in that period? (percentage)** *

## ENSURE Study - legislation

Questionnaire regarding AED use in all the european nations - EuropeaN Study about AED Use by layREscuers

19. **12. Is there any special legislation on the use of AEDs in your region and/or country today?** *

*Contrassegna solo un ovale.*

- Yes
- No
- Unknown

20. **13. Who is allowed to use an AED in your region?** *

*Contrassegna solo un ovale.*

- Everybody
- Everybody who has been trained
- Nurses
- Nurses / EMT / Paramedics
- Nurses / EMT / Paramedics with a special training
- Nurses / EMT / Paramedics under direct supervision of a physician
- Physicians only
- Unknown

21. **14. Has the legislation on the use of AEDs changed after December 2017?** *

*Contrassegna solo un ovale.*

- Yes, it was changed
- No, it is the same in force in December 2017

22. **15. The legislation on the use of AEDs is similar in your region respect to whole country?** *

*Contrassegna solo un ovale.*

- Is the same respect to whole country
- Is different in my region respect to whole country

## ENSURE Study

Please, refer to the period from October 1st, 2017 to December 31st, 2017

23. **16. How many AEDs were available for public access defibrillation (both in fixed positions and mobile) in your region/country in the study period?**

24. **17. There was a AEDs' mapping system in your region/country in the study period?** *

*Contrassegna solo un ovale.*

- Yes *Passa alla domanda 25.*
- Yes, only in some areas
- No *Passa alla domanda 25.*
- Unknown *Passa alla domanda 25.*

## Regarding question 17

Please, refer to the period from October 1st, 2017 to December 31st, 2017

25. **17.1 Which are the name of the areas and what is the number of inhabitants for each area in which there was a AEDs' mapping system in the study period?** *

## ENSURE Study

Please, refer to the period from October 1st, 2017 to December 31st, 2017

26. **18. Did the emergency response system (EMS) activate firefighters, police officers or other first responders equipped with an AED in order to improve the chance of early defibrillation in your region/country in the study period?** *

Please, consider that BLS ambulance crew is NOT considered as first responders

*Contrassegna solo un ovale.*

- Yes *Passa alla domanda 26.*
- Yes, only in some areas *Passa alla domanda 27.*
- No *Passa alla domanda 29.*
- Unknown *Passa alla domanda 29.*

*Passa alla domanda 29.*

## Regarding question 18

Please, refer to the period from October 1st, 2017 to December 31st, 2017

27. **18.1 Who was activated, equipped with an AED, by EMS in case of cardiac arrest in the study period?** *

*Seleziona tutte le voci applicabili.*

- [ ] Firefighters equipped with an AED
- [ ] Police officers equipped with an AED
- [ ] Other First Responders equipped with an AED
- [ ] Others

*Passa alla domanda 29.*

## Regarding question 18

Please, refer to the period from October 1st, 2017 to December 31st, 2017

28. **18.1 Who was activated, equipped with an AED, by EMS in case of cardiac arrest in the study period?** *
*Seleziona tutte le voci applicabili.*

- [ ] Firefighters equipped with an AED
- [ ] Police officers equipped with an AED
- [ ] Other First Responders equipped with an AED
- [ ] Others

## Regarding question 18

Please, refer to the period from October 1st, 2017 to December 31st, 2017

29. **18.2 Which are the name of the areas and what is the number of inhabitants for each area in which EMS activate that people (firefighters / police officers or other first responders) equipped with AED in the study period?** *

______________________________

______________________________

______________________________

______________________________

______________________________

## ENSURE Study

Please, refer to the period from October 1st, 2017 to December 31st, 2017

30. **19. Was there implemented a system to alert laypeople or first responders (not equipped with AED) to perform CPR before EMS arrival in case of out-of-hospital cardiac arrest in your region/country in the study period?** *
Please, consider that BLS ambulance crew is NOT considered as first responders
*Contrassegna solo un ovale.*

- ( ) Yes, a SMS system
- ( ) Yes, an APP system
- ( ) Yes, a SMS system and an APP system (depends of the areas)
- ( ) No *Passa alla domanda 33.*
- ( ) Unknown *Passa alla domanda 33.*

## Regarding question 19

Please, refer to the period from October 1st, 2017 to December 31st, 2017

31. **19.1 Does the system indicate the nearest AED available (in the study period)?** *
*Contrassegna solo un ovale.*

- ( ) Yes
- ( ) No

## Regarding question 19

Please, refer to the period from October 1st, 2017 to December 31st, 2017

32. **19.2 Is that system implemented in all your region/country or only in certain areas in the study period?** *

*Contrassegna solo un ovale.*

- In all areas *Passa alla domanda 33.*
- Only in certain areas

## Regarding question 19

Please, refer to the period from October 1st, 2017 to December 31st, 2017

33. **19.3 Which are the name of the areas and what is the number of inhabitants for each area in which EMS alert laypeople or first responders (not equipped with AED) to perform CPR before EMS arrival?** *

## ENSURE Study

34. **20. Were there any changes nowadays in first responder or laypeople activation by EMS compared to October-December 2017?** *

*Contrassegna solo un ovale.*

- Yes
- No

## ENSURE Study

35. **21. Further comments or suggestions** *

Powered by
Google Forms
